# Self-perception of aging and associated factors among older adults in Morocco

**DOI:** 10.1590/1980-5764-DN-2025-0316

**Published:** 2026-04-20

**Authors:** Hicham Mejdouli, Sana El Fadeli, Hakima Amor, Abdellatif Baali, Nadia Ouzennou

**Affiliations:** 1 Cadi Ayyad University Faculty of Sciences Semlalia, Biology. Bd. Prince My Abdellah, Marrakech, Morocco.; 2 Health Establishment Network Service, Provincial Delegation of the Ministry of Health and Social Protection, Essaouira, Morocco.; 3 Regional Health Directorate, Higher Institute of Nursing Professions and Health Techniques (ISPITS-M), Nursing Care Department, Marrakech, Morocco.

**Keywords:** Self Concept, Aging, Aged, Life Style, Health, Autoimagem, Envelhecimento, Idoso, Estilo de vida, Saúde

## Abstract

Self-perception of aging is considered one of the indicators of health and vulnerability. Understanding how older adults perceive their aging can be an effective way to develop strategies to prevent vulnerability and promote healthy aging.

**Objective:**

The aim of this study was to investigate the self-perception of aging and its associated factors in a group of older adults in Morocco to contribute to healthy aging.

**Methods:**

This was a cross-sectional study conducted between June 2022 and July 2023 among 496 people aged 60 years and over who visited health centers in the province of Essaouira. A positive or negative self-perception of aging was established using the Attitudes Toward Own Aging subscale, which includes five items from the Philadelphia Geriatric Center Morale Scale. Sociodemographic characteristics and data related to lifestyle and health status were collected through interviews, by consulting health records, and were supplemented with anthropometric measurements. Binary logistic regression was used to determine the potential factors influencing self-perception of aging.

**Results:**

A negative self-perception of aging was identified in 21.4% of participants. Multivariate analysis revealed that being unmarried (OR 2.027; 95%CI 1.261–3.259), having depressive symptoms (OR 1.729; 95%CI 1.082–2.765), and being dependent (OR 1.926; 95%CI 1.134–3.272) were the main factors statistically associated with a negative self-perception of aging.

**Conclusion:**

A strategy to prevent loneliness and social isolation, as well as screening programs for depression and dependency in older adults, could contribute to successful and healthy aging.

## RESUMO.

A auto-percepção do envelhecimento é considerada um dos indicadores de saúde e vulnerabilidade. Compreender como os idosos percebem o seu envelhecimento pode ser uma forma eficaz de desenvolver estratégias para prevenir a vulnerabilidade e promover um envelhecimento saudável.

**Objetivo:**

Este estudo teve como objetivo investigar a auto-percepção do envelhecimento e os fatores associados em um grupo de idosos no Marrocos, a fim de contribuir para o envelhecimento saudável.

**Métodos:**

Trata-se de um estudo transversal realizado entre junho de 2022 e julho de 2023, envolvendo 496 pessoas com idade igual ou superior a 60 anos que frequentaram centros de saúde na província de Essaouira. A auto-percepção positiva ou negativa do envelhecimento foi avaliada por meio da subescala Attitudes Toward Own Aging, que inclui cinco itens da Philadelphia Geriatric Center Morale Scale. As características sociodemográficas e os dados relacionados ao estilo de vida e ao estado de saúde foram coletados por meio de entrevistas, consulta aos registros de saúde e complementados por medidas antropométricas. A regressão logística binária foi utilizada para identificar os fatores potenciais que influenciam a auto-percepção do envelhecimento.

**Resultados:**

Uma auto-percepção negativa do envelhecimento foi identificada em 21,4% dos participantes. A análise multivariada revelou que não ser casado(a) (OR 2,027; IC95% 1,261–3,259), apresentar sintomas depressivos (OR 1,729; IC95% 1,082–2,765) e ser dependente (OR 1,926; IC95% 1,134–3,272) foram os principais fatores estatisticamente associados a uma auto-percepção negativa do envelhecimento.

**Conclusão:**

Estratégias de prevenção da solidão e do isolamento social, bem como programas de rastreamento da depressão e da dependência em idosos, podem contribuir para um envelhecimento bem-sucedido e saudável.

## INTRODUCTION

 In Morocco, the aging of the population is an established fact^1^. The number of older adults has increased from one million to 4.5 million between 1970 and 2022, with an increase of an annual rate of 2.8%, higher than that of the entire population of Morocco, which stands at 1.7%. By 2050, the demographic weight of older adults will be 23.2% instead of 12.2% currently^2^. 

 The rapid and accelerated aging of the Moroccan population is accompanied by health problems and chronic diseases that constitute a major challenge for health professionals and medico-social institutions, but also for the older adults and their loved ones^3^. The results of the latest National Population and Family Health Survey revealed that 64.4% of people aged 60 years and over are affected by at least one chronic disease^4^. The national survey on disability in Morocco, for its part, indicated that the prevalence of disability is higher among people aged 60 years and over (33.7%) compared to other age groups due to certain diseases and pathologies that lead to disability and dependency^5^. 

 Furthermore, there is growing evidence that key health-related behaviors can exert a strong influence on older adults’ abilities^6^. A healthy lifestyle is an important factor in successful aging and helps prevent cognitive and functional decline associated with the aging process^7,8^. 

 Self-perception of aging is also considered one of the indicators of health and vulnerability^9,10^. Indeed, the more negatively individuals perceive aging, the greater the risk of reaching a concerning state of vulnerability^11^. Therefore, understanding how older people perceive their aging can be an effective way to develop strategies to prevent vulnerability and promote healthy aging. The challenge is therefore to promote well-being and improve the quality of life of Moroccan older adults in a context of rapid aging where the older adults are vulnerable, and the social protection system is still fragile^12^. 

 Thus, the objective of this study is to assess the self-perception of aging among older adults in Morocco and to identify influencing factors in order to contribute to healthy aging. 

## METHODS

### Ethics approval and consent to participate

 The study was conducted after obtaining authorization from the health authorities and the Ethics Committee of the Moroccan Association for Research and Ethics (Letter of Approval: 8/REC/21). All participants provided informed consent before taking part in the study, and interviews were conducted after they had completed the medical consultation or service for which they visited the health center. 

### Design of the study

 This was a cross-sectional study, using a quantitative research method, which aimed to assess the self-perception of aging among older adults in Morocco and to identify influencing factors contributing to healthy aging. 

 The target population was people aged 60 years and over in the province of Essaouira. This province is part of the Marrakech-Safi region, which is located in central Morocco. The province is divided into 57 municipalities, of which 52 are rural and 5 urban. The total population of the province is 449133, of whom 12.8% are aged 60 years and over^13^. 

 We recruited 496 individuals aged 60 years and older who frequented health centers. The health centers involved in this study were primary healthcare facilities belonging to the public health system. They provide first-level preventive and curative services, including medical consultations, follow-up of chronic diseases, and maternal and child health care. Older adults in the province of Essaouira visit these centers regularly for routine health monitoring or medical consultations, which facilitated participant recruitment during their visits. A stratified random sampling method with proportional allocation was adopted to identify the number of participants per center, based on the number of older adults served by each health facility. This approach made it possible to extract from each stratum representing the older adults, a number proportional to their representation in the target population of the 20 health centers that were the study sites. Participants were randomly selected during their visits to the health centers until the number per center was reached. 

 Exclusion criteria included people with severe psychiatric and/or neurological disorders. 

### Data collection

 The data were collected between June 2022 and July 2023, at the health centers of the province of Essaouira through face-to-face interviews with the older adults, using a structured questionnaire, which was supplemented by anthropometric measurements (weight and height). 

 In order not to influence the participants’ responses, the interviewer was not a member of the health center’s medical team. 

### Variables and modalities

 Sociodemographic and socioeconomic characteristics were collected using the questionnaire. The variables included gender, age (which was identified using the participant’s national identity card), marital status (married or not), family structure (single, nuclear family, or extended family), residential area (urban or rural), education level (literate or illiterate), occupational status (without profession, retired, or active at the time of the survey), medical coverage (with or without coverage), and income (below or above the Guaranteed Interprofessional Minimum Wage (GIMW)). The value of the GIMW in Morocco is 2698 dirhams per month in 2022, or 272.68 USD^14^. 

 Lifestyle variables included physical activity and smoking. Physical activity was estimated based on the weekly physical activity duration. Older adults were categorized into two groups based on reported duration, following the WHO recommendations for older adults^15^. The first group consisted of individuals with insufficient physical activity (less than 150 minutes per week), and the second group included people with sufficient physical activity (more than 150 minutes per week). 

 The assessment of tobacco consumption was conducted according to the subjects’ answers to the following question (yes or no): "Do you use tobacco?" 

 Participants’ health status data included subjective health, depressive symptoms, chronic illnesses, nutritional status, and autonomy. 

 Subjective health status was assessed through the following question: "How do you feel?", a question that has two response options: "In good health, In poor health." 

 Depressive symptoms were identified using the short form of the Geriatric Depression Scale (GDS-15). An individual is considered to have depressive symptoms if their score is equal to or higher than 6, while a score below 6 indicates the absence of depressive symptoms^16^. 

 Medically diagnosed chronic diseases were identified using the health records of the subjects studied. 

 Nutritional status was assessed by calculating the Body Mass Index (BMI). According to the WHO classification for the older adults^17^:

- Underweight: BMI below 18.5 (below 21 for people aged 71 years and over);
- Normal weight: BMI between 18.5 and 24.9 (between 21 and 24.9 for people aged 71 years and over);
- Overweight: BMI≥25 (excess weight is a BMI between 25 and 29.9, and obesity is defined as BMI≥30).

Weight was measured using an electronic scale. Height was measured using a height rod for individuals able to stand and was estimated from leg height using a tape measure for those unable to stand. Height estimation by leg height was done using the following equations^18^:

- For men: height (cm)=84.88–0.24 x age (years)+1.83 x leg height (cm);
- For women: height (cm)=64.19–0.40 x age (years)+2.02 x leg height (cm).

The autonomy of the subjects studied was assessed using the Katz index (ADL—activities of daily living scale), which allows for the evaluation of the functional status and the ability of older adults to carry out daily activities independently^19^. The Katz index ranks performance in six activities: "grooming," "dressing," "toilet," "locomotion," "continence," and "feeding." The score on this scale ranges from 0 to 6. The older adults is considered:

- Totally dependent: score=0
- Partially dependent: score between 1 and 5
- Independent (completely autonomous): score=6

In this study, we subdivided the participants into two groups, namely the independent group (complete autonomy) and the dependent group (partial or total dependence). 

 The Attitude Toward Own Aging subscale, which includes five items of the Philadelphia Geriatric Center Morale Scale^20^ were used to assess self-perception of aging. The five items are:

- As I get older, things get worse (agree = 1; disagree = 0);
- I have the same amount of energy as I did last year (agree = 0; disagree = 1);
- As we get older, we become less useful (agree = 1; disagree = 0);
- I am happier now than when I was younger (agree = 0; disagree = 1);
- Health-wise, things are (0 = better, 1 = worse, or expected).

Adding the scores of the five items gives a total score ranging from 0 to 5; a higher score reflects a more negative perception. According to the classification used by Moser et al.^21^, we categorized the total score of self-perception of aging into:

- Positive (values 0–2)
- Negative (values 3–5).

### Statistical analysis

 Data were examined using IBM’s Statistical Package for the Social Sciences (SPSS) version 25. Cronbach’s alpha coefficient was utilized to assess the internal consistency of the items on the Attitude Toward Own Aging subscale. The ꭓ^2^ test was used to study the association between the self-perception of aging among the group studied and the other variables retained in the present study. Binary logistic regression was applied to eliminate confounding factors and capture the weight of associated variables. Statistical significance was defined as p<0.05. 

## RESULTS

 The study included 496 individuals aged 60 years and older, including 270 men (54.4%). The age of the participants ranged from 60 to 95 years, with an average of 69.6 years (±7.7), and the 60–69 age group was the most represented (56.5%). Overall, the majority of participants were married (68.5%), lived with their family (92.5%, including 60.7% in nuclear families and 31.8% in extended families), were of rural origin (50.8%), and were illiterate (78.2%). 

 On the socioeconomic level, one in two participants was employed at the time of the survey, and almost half of the subjects surveyed (49.4%) had an income below the minimum wage. Moreover, the majority (76.4%) had medical coverage. 

 The main sociodemographic and socioeconomic characteristics of the studied group are given in Table 1. 

**Table 1:** Sociodemographic and socioeconomic characteristics of participants.

Variables		n	%
Sex			
	Men	270	54.4
	Women	226	45.6
Age groups (years)			
	60–69	280	56.5
	70–79	153	30.8
	≥80	63	12.7
Marital status			
	Unmarried	156	31.5
	Married	340	68.5
Family structure			
	Living alone	37	7.5
	Nuclear family	301	60.7
	Extended family	158	31.8
Place of residence			
	Urban	244	49.2
	Rural	252	50.8
Education			
	Illiterate	388	78.2
	Literate	108	21.8
Socio-professional activity			
	Unemployed	171	34.5
	Retired	77	15.5
	Employed	248	50.0
Income			
	<GIMW	245	49.4
	≥GIMW	251	50.6
Medical coverage			
	Yes	379	76.4
	No	117	23.6

Abbreviation: GIMW, Guaranteed Interprofessional Minimum Wage.

### Lifestyle and health

Table 2 describes the lifestyle and health variables of the older adults studied. It shows that almost three-quarters of the participants (77.6%) were sedentary and 10.7% were smokers. 

**Table 2:** Lifestyle and health status of participants.

Variables		n (%)
Physical activity		
	Sufficient	111 (22.4)
	Insufficient	385 (77.6)
Smoking		
	Yes	53 (10.7)
	No	443 (89.3)
Subjective health status		
	Good	366 (73.8)
	Wrong	130 (26.2)
Depressive symptoms		
	Yes	176 (35.4)
	No	320 (64.5)
Chronic illness		
	Yes	276 (55.6)
	No	220 (44.4)
Hypertension		
	Yes	170 (34.3)
	No	326 (65.7)
Diabetes		
	Yes	131 (26.4)
	No	365 (73.6)
Cardiovascular diseases		
	Yes	46 (9.2)
	No	450 (90.8)
Joint diseases		
	Yes	43 (8.7)
	No	453 (91.3)
Kidney diseases		
	Yes	17 (3.4)
	No	479 (96.6)
Respiratory diseases		
	Yes	16 (3.2)
	No	480 (96.8)
BMI categories		
	Underweight	45 (9.1)
	Normal weight	270 (54.4)
	Overweight	181 (36.5)
Autonomy		
	Independent	314 (63.3)
	Dependent	182 (36.7)

Abbreviation: BMI, Body Mass Index.

 Regarding health status, 73.8% of the participants perceived themselves to be in good health. Depressive symptoms were detected in 35.4% of the participants. 

 The majority of participants (55.6%) had at least one chronic disease diagnosed by medical personnel. Given that the same person could have several diseases, hypertension was the most common chronic condition among the older adults studied, with a prevalence of 34.3%, followed by diabetes (26.4%). 

 A study of participants’ nutritional status according to BMI showed that 9.1% were underweight and 36.5% were overweight, including 7.7% who were obese. 

 An assessment of autonomy using the ADL scale revealed that the majority of participants (63.3%) were autonomous, while 36.7% were totally or partially dependent (with 0.6% being totally dependent). 

 For our sample as a whole, the self-perception of aging score ranged from 1 to 5, with an average of 1.5±1.4. Cronbach’s alpha coefficient was 0.78, indicating good internal consistency. Thus, a negative self-perception of aging was identified in 21.4% of the participants. Table 3 shows participants’ responses to the five items of the Attitudes Toward Own Aging subscale. 

**Table 3:** Distribution of participants according to their responses to the Attitudes Toward Own Aging subscale.

Items	Agree (%)	Disagree (%)
As I get older, things get worse	20.6	79.4
I have the same amount of energy as last year	53.0	47.0
As people age, we become less useful	23.6	76.4
I am happier now than when I was younger	78.8	21.2
Health-wise, things are better	71.8	28.2

 The results of the study of the relationship between self-perception of aging and the sociodemographic and health variables of older adults are given in Table 4. According to this table, self-perception of aging appears to be statistically associated with marital status, family structure, income, physical activity, subjective health status, BMI category, and autonomy. 

**Table 4:** Self-perception of aging and sociodemographic and health variables.

Variables		n	Self-perception of aging		p-value
			Positive (%)	Negative (%)	
Sex					
	Men	270	80.0	20.0	0.240*
	Women	226	77.0	23.0	
Age groups (years)					
	60–69	280	81.1	18.9	0.144*
	70–79	153	73.2	26.8	
	≥80	63	81.0	19.0	
Marital status					
	Unmarried	156	67.9	32.1	0.001†
	Married	340	83.5	16.5	
Family structure					
	Living alone	37	62.2	37.8	0.034‡
	Nuclear family	301	80.7	19.3	
	Extended family	158	78.5	21.5	
Place of residence					
	Urban	244	77.5	22.5	0.303*
	Rural	252	79.8	20.2	
Education					
	Illiterate	388	79.9	20.1	0.121*
	Literate	108	74.1	25.9	
Socio-professional activity					
	Unemployed	171	74.9	25.1	0.317*
	Retired	77	81.8	18.2	
	Employed	248	80.2	19.8	
Income					
	<GIMW	245	73.5	26.5	0.004†
	≥GIMW	251	83.7	16.3	
Medical coverage					
	Yes	379	78.4	21.6	0.453*
	No	117	79.5	20.5	
Physical activity					
	Sufficient	111	84.7	15.3	0.048‡
	Insufficient	385	76.9	23.1	
Smoking					
	Yes	53	77.4	22.6	0.465*
	No	443	78.8	21.2	
Subjective health status					
	Good	366	82.0	18.0	0.002†
	Wrong	130	69.2	30.8	
Depressive symptoms					
	Yes	176	82.8	17.2	0.002†
	No	320	71.0	29.0	
Chronic illness					
	Yes	276	78.6	21.4	0.543*
	No	220	78.6	21.4	
BMI categories					
	Underweight	45	60.0	40.0	0.006†
	Normal weight	270	79.9	20.1	
	Overweight	181	81.3	18.7	
Autonomy					
	Independent	314	84.7	15.3	0.001†
	Dependent	182	68.1	31.9	

Abbreviations: GIMW, Guaranteed Interprofessional Minimum Wage, BMI, Body Mass Index.

Notes: *p<0.05 (statistically significant); †p<0.01 (highly significant); ‡p<0.001 (very highly significant).

 In order to account for the various confounding factors and to highlight the impact of each of the explanatory variables retained on the dependent variable, which is self-perception of aging, we applied the binary logistic regression model (Table 5). The results of this analysis show that only the variables marital status, depressive symptoms, and autonomy are statistically associated with a negative self-perception of aging. 

**Table 5:** Adjusted odds ratio adjusted for negative self-perception of aging and sociodemographic and health factors.

Variables	β	p-value	OR (95%CI)
Marital status	0.707	0.004*	2.027 (1.261–3.259)
Family structure	0.236	0.208†	1.266 (0.877–1.829)
Income	0.293	0.220†	1.341 (0.840–2.141)
Physical activity	0.419	0.169†	1.520 (0837–2.761)
Subjective health status	0.050	0.726†	1.052 (0.794–1.392)
Depressive symptoms	0.548	0.022‡	1.729 (1.082–2.765)
BMI categories	-0.134	0.295†	0.875 (0.681–1.123)
Autonomy	0.656	0.015‡	1.926 (1.134–3.272)

Abbreviations: β, Coefficient; OR, Odds ratio; CI, confidence interval.

Notes: *p<0.01 (highly significant); †p≥0.05 (not significant); ‡p<0.05 (statistically significant);

## DISCUSSION

 The aim of this study was to assess the self-perception of aging among older adults in Morocco and to identify influencing factors. 

 The main results of this study revealed that the lifestyles of the older adults studied were marked by a sedentary lifestyle, with only 22.4% reporting sufficient physical activity. These results corroborate those of the National Survey on Population and Family Health, which indicated that the majority of older adults in Morocco are sedentary, with only 23.7% engaging in regular physical activity^4^. The proportion of smokers was 10.7%, slightly lower than that reported in the National Survey on Common Risk Factors for Non-Communicable Diseases, where the prevalence of all forms of tobacco use was 13.4% in the general population^22^. This discrepancy could be explained by the adoption of a healthier lifestyle with advancing age. 

 In terms of health status, self-perceived health is a good indicator of people’s general health, both physical and mental^23^. In our sample, 73.8% of the subjects studied perceived themselves to be in good health, which confirms the results of many previous studies indicating that the majority of older adults in Morocco have a positive perception of their state of health^12,24,25^. However, depressive symptoms were identified in 35.5% of participants. This prevalence of depressive symptoms, assessed using the GDS-15, is lower than that reported in a recent study conducted in the city of Marrakech (central Morocco), which found 50% prevalence using the same measurement tool^26^. This discrepancy could be explained by the socioeconomic and environmental differences between the city of Marrakech and the province of Essaouira^27^. The city of Marrakech is primarily urban, while the province of Essaouira is mostly rural. Socioeconomic level and living conditions play an important role in the susceptibility of older adults to depression^28,29^. On the whole, however, it seems that depressive symptoms are highly prevalent among Moroccan seniors and tend to be neglected, which calls for a systematic strategy of screening and managing depression in health establishments and at the community level. 

 The majority of those surveyed (55.6%) had at least one medically diagnosed chronic disease. These results are in line with data from the Ministry of Health and Social Protection, which reported that more than half of people aged 60 years and over in Morocco suffer from at least one chronic condition^4^. The nutritional status of the group studied was characterized by the double burden of malnutrition, with both underweight (9.1%) and overweight (36.5%) being observed. This nutritional profile is almost identical to that of populations in neighboring countries. An Algerian study revealed that 11.3% of older adults in Algeria are underweight^29^ while a Tunisian study showed that 43.4% of Tunisians aged 60 years and over were overweight^30^. Although BMI was used as an indicator of nutritional status in this study, other tools (e.g., MNA) could provide a more comprehensive and accurate assessment in older adults. 

 With regard to autonomy, the results showed that 36.7% of participants were either totally or partially dependent. This proportion is 30.4% for the city of Marrakech^26^. It is clear that dependence can occur gradually and progressively, or suddenly at the extreme stages of functional decline^31^, hence the need for early detection. 

 The study of self-perception of aging showed that the majority (78.6%) of older people have a positive self-perception of aging, while 21.4% perceive it negatively. These results are consistent with those of a Swiss study, which also used the five items of the Attitudes Toward Own Aging subscale, and revealed that the majority of older people viewed their aging positively^32^. 

 Binary logistic regression analysis showed that only the variables marital status, depressive symptoms, and autonomy were significantly and independently associated with negative self-perception of aging. Indeed, the results showed that being married was associated with a more positive self-perception of aging, which is consistent with a large body of literature that has demonstrated that married older adults generally have a better perception of their aging^33,34,35^. Spouses play a particularly important role in reducing risk factors such as social isolation and loneliness, as well as improving mental health, life satisfaction, and overall quality of life^36^. 

 The results also showed that depressive symptoms were more prevalent among subjects with a negative self-perception of aging. This result was expected insofar as the perception of aging is one of the indicators of health and vulnerability^9,10^. Our results are thus consistent with those of a recent U.S. study, which indicated that a negative self-perception of aging is linked to increased depressive symptoms^37^. 

 Self-perception of aging and autonomy were also found to be statistically associated. Individuals in dependent situations had a more negative self-perception of aging. Previous research has shown that a negative self-perception of aging can predict disability in the older adults, as well as the onset of difficulties in performing activities of daily living^21^. Moreover, maintaining good physical health increases an older adults ability to live independently, to carry out activities of daily living, and to perform other self-care activities and housework, which supports autonomy and contributes to psychological well-being^38^. 

 This study acknowledges the limitations of using BMI as a simple yet less accurate indicator of the nutritional status of older adults compared to other tools. Furthermore, the generally positive self-perception of aging observed among participants may be influenced by the rural lifestyle, strong social cohesion, and cultural valorization of elderhood. Age-related resilience factors, together with lower exposure to urban stressors, may contribute to enhanced emotional well-being and a favorable perception of aging despite limited material resources. 

 In conclusion, a considerable proportion of older people exhibited a negative self-perception of aging. This self-perception is influenced by multiple factors, including marital status, depressive symptoms, and loss of autonomy. 

 This study highlights the impact of partner absence on the psychological well-being of the older adults. Hence the need to implement measures to combat loneliness and social isolation in this population. Given the high prevalence of depressive symptoms, the introduction of a systematic depression screening program in primary care establishments is strongly recommended. Furthermore, the results suggest that a negative self-perception of aging could serve as an early indicator of functional decline, allowing for better identification of at-risk individuals, delaying the progression of dependency, and extending the period of independent living. 

## Data Availability

The datasets generated and/or analyzed during the current study are available from the corresponding author upon reasonable request.

## References

[1] Conseil économique, social et environnemental (2015). Les personnes âgées au Maroc [Internet].

[2] Haut-Commissariat au Plan du Royaume du Maroc (2022). Note d’information à l’occasion de la journée internationale des personnes âgées [Internet].

[3] Organisation mondiale de la Santé (2024). Vieillissement et santé [Internet].

[4] Ministère de la Santé et de la Protection Sociale (2018). Enquête Nationale sur la Population et la Santé Familiale Maroc [Internet].

[5] Ministère de la Famille, de la Solidarité, de l’Egalité et du Développement Social (2014). Enquête nationale sur le Handicap – Rapport détaillé [Internet].

[6] Organisation Mondiale de la Santé (2016). Stratégie et plan d’action mondiaux sur le vieillissement et la santé 2016–2020 : vers un monde où chacun puisse vivre longtemps et en bonne santé [Internet].

[7] Renner B, Spivak Y, Kwon S, Schwarzer R (2007). Does age make a difference? Predicting physical activity of South Koreans. Psychol Aging.

[8] Paterson DH, Warburton DE (2010). Physical activity and functional limitations in older adults: a systematic review related to Canada’s Physical Activity Guidelines. Int J Behav Nutr Phys Act.

[9] Hausdorff JM, Levy BR, Wei JY (1999). The power of ageism on physical function of older persons: reversibility of age-related gait changes. J Am Geriatr Soc.

[10] Levy BR, Slade MD, Kunkel SR, Kasl SV (2002). Longevity increased by positive self-perceptions of aging. Pers Soc Psychol.

[11] de Bruin A, Picavet HS, Picavet A (1996). Health interview surveys. Towards international harmonization of methods and instruments. WHO Reg Publ Eur Ser.

[12] Baali A, Amor H, Zakaria R, El Khoudri N, Fathi N, El Kadmiri N (2021). Assessment of the quality of life of a group of subjects aged 65 and over Morocco. Anthropol Res Stud.

[13] Centre d’Etudes et de Recherches Démographiques (2017). Projections de la population des régions et des provinces 2014-2030. [Internet].

[14] Manoussi A, Nacer N, Kajjoune I, Baali A, Amor H, Ouzennou N (2025). Prevalence and predictors of overweight and obesity among women of childbearing age in the province of Essaouira, Morocco. BMC Public Health.

[15] Organisation mondiale de la Santé (2010). Recommandations mondiales sur l’activité physique pour la santé [Internet].

[16] Yesavage JA, Sheikh JI (1986). 9/Geriatric Depression Scale (GDS). Clin Gerontol.

[17] World Health Organization (2021). Obesity and overweight [Internet].

[18] Chumlea WC, Roche AF, Steinbaugh ML (1985). Estimating stature from knee height for persons 60 to 90 years of age. J Am Geriatr Soc.

[19] Katz S, Dowth TD, Cash HR, Grotz RC (1970). Progress in the development of the index of ADL. Gerontologist.

[20] Lawton MP (1975). The Philadelphia Geriatric Center Morale Scale: a revision. J Gerontol.

[21] Moser C, Spagnoli J, Santos-Eggimann B (2011). Self-perception of aging and vulnerability to adverse outcomes at the age of 65-70 years. J Gerontol B Psychol Sci Soc Sci.

[22] Ministère de la Santé (2018). Enquête nationale sur les facteurs de risque communs des maladies non transmissibles 2017-2018: rapport [Internet].

[23] Deeg DJH, Bath PA (2003). Self-rated health, gender, and mortality in older persons: introduction to a special section. Gerontologist.

[24] Mejdouli H, Baali A, Amor H, Ouzennou N (2024). Prevalence and determinants of frailty among the elderly in the province of Essaouira, Morocco. Pan Afr Med J.

[25] Mejdouli H, Baali A, Ouzennou N, Amor H (2023). Qualité de vie et estime de soi des personnes âgées dans la province d’Essaouira au Maroc: état des lieux et facteurs influençant. NPG Neurol Psychiatr Gériatrie.

[26] El Harsi EM, Izel O, Benksim A, Cherkaoui M (2023). Factors influencing older adults’ satisfaction with caregivers’ communication. Dement Neuropsychol.

[27] Haut-Commissariat au Plan (2024). Indicateurs démographiques et socioéconomiques du royaume du Maroc selon les résultats du recensement général de 2024 [Internet].

[28] Gallarda T, Lôo H (2009). Dépression et personnes âgées. L’Encéphale.

[29] Menadi N, Khaled M, Merrakchi B, Belbraouet S (2013). «Nutritional Status of Elderly People Living at Home in Sidi-Bel-Abbes (West Algeria),». Food and Nutrition Sciences.

[30] Khelil M, Ben Abdelaziz A, Zanina Y, Yahia F, Ben Hassine D, Ben Rejeb N (2022). Epidemiology of obesity in Tunisia: HSHS4 study (Hammam Sousse Sahloul Heart Study). Tunis Med.

[31] Hoertel N, Le Strat Y, Gorwood P, Béra-Potelle C, Schuster JP, Manetti A (2013). Why does the lifetime prevalence of major depressive disorder in the elderly appear to be lower than in younger adults? Results from a national representative sample. J Affect Disord.

[32] Rendall MS, Weden MM, Favreault MM, Waldron H (2011). The protective effect of marriage for survival: a review and update. Demography.

[33] Fried LP, Kronmal RA, Newman AB, Bild DE, Mittelmark MB, Polak JF (1998). Risk factors for 5-year mortality in older adults: the Cardiovascular Health Study. JAMA.

[34] Jang SN, Kawachi I, Chang J, Boo K, Shin HG, Lee H (2009). Marital status, gender, and depression: analysis of the baseline survey of the Korean Longitudinal Study of Ageing (KLoSA). Soc Sci Med.

[35] Kavirajan H, Vahia IV, Thompson WK, Depp C, Allison M, Jeste DV (2011). Attitude toward own aging and mental health in post-menopausal women. Asian J Psychiatr.

[36] Organisation mondiale de la Santé (2025). Santé mentale et lien social [Internet].

[37] Komalasari R, Hill NL, Berish D, Mogle J (2024). More positive attitudes to aging are associated with lower subjective cognitive decline: moderating roles of affective well-being. J Appl Gerontol.

[38] Celik SS, Celik Y, Hikmet N, Khan MM (2018). Factors affecting life satisfaction of older adults in Turkey. Int J Aging Hum Dev.

